# Hereditary sensory neuropathy type 1-associated deoxysphingolipids cause neurotoxicity, acute calcium handling abnormalities and mitochondrial dysfunction *in vitro*

**DOI:** 10.1016/j.nbd.2018.05.008

**Published:** 2018-09

**Authors:** Emma R. Wilson, Umaiyal Kugathasan, Andrey Y. Abramov, Alex J. Clark, David L.H. Bennett, Mary M. Reilly, Linda Greensmith, Bernadett Kalmar

**Affiliations:** a Sobell Department of Motor Neuroscience and Movement Disorders, UCL Institute of Neurology, Queen Square, London WC1N 3BG, UK; b MRC Centre for Neuromuscular Diseases, UCL Institute of Neurology, Queen Square, London WC1N 3BG, UK; c Department of Molecular Neuroscience, UCL Institute of Neurology, Queen Square, London WC1N 3BG, UK; d Neural Injury Group, Nuffield Department of Clinical Neurosciences, University of Oxford, Oxford, UK

**Keywords:** ∆ψ_m_, mitochondrial membrane potential, CHOP, C/EBP homologous protein, CsA, cyclosporine A, DMSp, deoxymethylsphinganine, DRG, dorsal root ganglia, DSp, deoxysphinganine, ER, endoplasmic reticulum, FCCP, carbonyl cyanide-*p*-trifluoromethoxyphenylhydrazone, GADD153, growth arrest and DNA damage-inducible protein, HSN, hereditary sensory neuropathy, MAM, mitochondria-associated ER membrane, MN, motor neuron, mPTP, mitochondrial permeability transition pore, NCX, sodium-Ca^2+^ exchanger, NMDA, *N*-methyl-D-asparatate, PLP, pyridoxal 5′-phosphate, PMCA, plasma membrane Ca^2+^ ATPase, SERCA, sarco/endoplasmic reticulum Ca^2+^-ATPase, SOC, store-operated calcium, Sp, sphinganine, SPT, serine palmitoyltransferase, TMRM, tetramethylrhodamine methyl ester, UPR, unfolded protein response, Sphingolipid, Deoxysphingolipid, Neuron, Peripheral neuropathy, ES-285, Mitochondria, Endoplasmic reticulum

## Abstract

Hereditary sensory neuropathy type 1 (HSN-1) is a peripheral neuropathy most frequently caused by mutations in the *SPTLC1* or *SPTLC2* genes, which code for two subunits of the enzyme serine palmitoyltransferase (SPT). SPT catalyzes the first step of *de novo* sphingolipid synthesis. Mutations in SPT result in a change in enzyme substrate specificity, which causes the production of atypical deoxysphinganine and deoxymethylsphinganine, rather than the normal enzyme product, sphinganine. Levels of these abnormal compounds are elevated in blood of HSN-1 patients and this is thought to cause the peripheral motor and sensory nerve damage that is characteristic of the disease, by a largely unresolved mechanism. In this study, we show that exogenous application of these deoxysphingoid bases causes dose- and time-dependent neurotoxicity in primary mammalian neurons, as determined by analysis of cell survival and neurite length. Acutely, deoxysphingoid base neurotoxicity manifests in abnormal Ca^2+^ handling by the endoplasmic reticulum (ER) and mitochondria as well as dysregulation of cell membrane store-operated Ca^2+^ channels. The changes in intracellular Ca^2+^ handling are accompanied by an early loss of mitochondrial membrane potential in deoxysphingoid base-treated motor and sensory neurons. Thus, these results suggest that exogenous deoxysphingoid base application causes neuronal mitochondrial dysfunction and Ca^2+^ handling deficits, which may play a critical role in the pathogenesis of HSN-1.

## Highlights

• Mutation in *SPTLC1* or *SPTLC2* are the most common cause of HSN-1.
• Abnormal sphingolipids produced by the mutant SPTLC1/2 enzyme cause neuronal death.
• Early targets of abnormal sphingolipids are the mitochondria and ER.
• Mitochondrial damage manifests in membrane potential loss and abnormal Ca^2+^ loading.
• Abnormal sphingolipids cause cell membrane leakage of Ca^2+^ through SOC channels.

## Graphical abstract

Graphical illustration demonstrating the proposed pathomechanism of the toxic, atypical deoxysphinganine and deoxymethylsphinganine, produced by mutant SPT in HSN-1.

The toxic sphingoid bases DSp and DMSp (or downstream metabolites) are released into the extracellular space where they destabilize neuronal cell membranes, resulting in dysfunction of neuronal ion channels such as store-operated Ca^2+^ (SOC) channels, upon stimulation. This results in elevated Ca^2+^ entry when SOC channels open, which is buffered by mitochondria. This increase in mitochondrial Ca^2+^ uptake in turn results in mitochondrial dysfunction, leading to loss of mitochondrial membrane potential and ultimately dysfunction of cellular processes and neuronal degeneration. (DSp: deoxysphinganine; DMSp: deoxymethylsphinganine).

**Figure:**
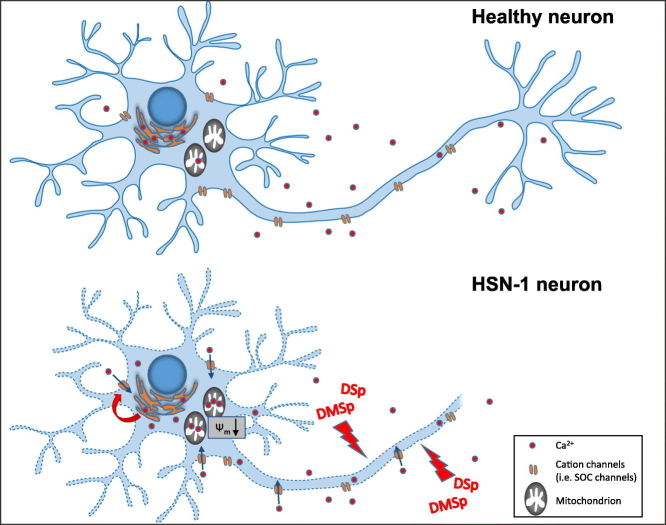

## 1 Introduction

Hereditary sensory neuropathy (HSN) is a group of inherited peripheral nerve disorders, the most common of which, HSN-1, is an autosomal, dominant disease frequently caused by mutations in the genes encoding serine palmitoyl transferase long chain base subunits 1 and 2 (*SPTLC1* and *SPTLC2*; Dawkins et al., 2001; Nicholson et al., 1996; Rotthier et al., 2010). HSN-1 is a slowly progressive neuropathy leading to profound loss of sensation, notably of pain and temperature (Houlden et al., 2004; Houlden et al., 2006). Despite its classification as a sensory neuropathy, HSN-1 is also associated with variable motor involvement, ranging from non-existent to severe. Similarly, there is significant diversity in the clinical presentation and age of disease onset, spanning the second to fifth decades of life (Auer-Grumbach, 2013; Davidson et al., 2012; Houlden et al., 2006; Reilly, 2007; Rotthier et al., 2012). Symptoms present in the distal limbs, which spread proximally in a progressive manner. As a result of perturbed sensation, most patients develop complications, such as recurrent ulcers and osteomyelitis, making amputations commonplace (Reilly, 2007). In addition to the characteristic, progressive loss of sensation, many HSN-1 patients frequently experience episodes of spontaneous, lancinating, burning pain (Houlden et al., 2004).

The ubiquitously expressed *SPTLC1* (9q22.1–22.3) and *SPTLC2* (14q24.3) genes encode two of the three major subunits of the enzyme serine palmitoyltransferase (SPT, Dawkins et al., 2001; Nicholson et al., 1996; Rotthier et al., 2010). To date, seven HSN-1-causing missense mutations in *SPTLC1* (Duan and Merrill Jr, 2015; Mroczek et al., 2015) and seven disease-causing mutations in *SPTLC2* have been identified (Duan and Merrill Jr, 2015; Mroczek et al., 2015; Suriyanarayanan et al., 2016). However, despite extensive screening no disease-causing mutations have been found in the third, *SPTLC3*-encoded, subunit of SPT (Rotthier et al., 2010). Clinically, patients with mutations in *SPTLC1* or *SPTLC2* are phenotypically indistinguishable and mutations in both genes have been suggested to cause HSN-1 *via* the same mechanism of dysregulated sphingolipid metabolism (Rotthier et al., 2012).

SPT is a pyridoxal 5′-phosphate (PLP)-dependent enzyme located on the outer membrane of the endoplasmic reticulum (ER, Hanada, 2003). It catalyzes the first and rate-limiting step committing substrates to *de novo* sphingolipid synthesis, *i.e.* the condensation of l-serine with palmitoyl-coenzyme A (see Supplementary Fig. S1, Buede et al., 1991; Hanada et al., 1997; Weiss and Stoffel, 1997). Sphingolipids and their derivatives are not only important structural elements in cell membranes, but also play a central role in signal transduction of external stimuli towards the nucleus, mediating cell differentiation, migration and apoptosis (Fyrst and Saba, 2010). Mutations in numerous enzymes within the sphingolipid pathway have been associated with neurodegeneration (Kolter and Sandhoff, 2006).

It has previously been shown that HSN-1 mutations alter the substrate specificity of the SPT enzyme, shifting its preference from the normal substrate serine to alanine or glycine. The subsequent reactions produce atypical sphingoid bases: deoxysphinganine (DSp) and deoxymethylsphinganine (DMSp), respectively (see Supplementary Fig. S1B and C, Gable et al., 2010; Penno et al., 2010; Zitomer et al., 2009). These deoxy-compounds are metabolised to form deoxyceramide and deoxymethylceramide, which lack the C1 hydroxyl group which is essential for further metabolism into the canonical complex sphingolipids, or indeed for degradation as seen in the typical sphingolipid pathway (Alecu et al., 2016a; Michel et al., 1997; Steiner et al., 2016). Elevated levels of deoxysphingoid bases have been detected in HSN-1 patient lymphoblasts and plasma as well as plasma of transgenic mice expressing mutant *SPTLC1* (Auer-Grumbach et al., 2013; Garofalo et al., 2011; Murphy et al., 2013; Penno et al., 2010; Rotthier et al., 2011; Suriyanarayanan et al., 2016). Interestingly, elevated levels have also been detected in the plasma of patients with metabolic syndrome and type 2 diabetes. Blood deoxysphingolipid levels are also elevated in animal models of diabetes, for example in the streptozotocin-induced rat diabetes model and in the leptin-deficient *ob*/*ob* mouse (Bertea et al., 2010; Othman et al., 2014; Othman et al., 2012; Zuellig et al., 2014). A common complication of diabetes is diabetic neuropathy, which presents clinically in a very similar manner to HSN-1 (Callaghan et al., 2012). In addition, cells deprived of serine have also been shown to generate elevated levels of deoxysphingoid bases (Esaki et al., 2015).

Results from previous studies have suggested that these abnormal deoxysphingoid bases are harmful to cells, as shown in a number of *in vitro* models (Cuadros et al., 2000; Esaki et al., 2015; Penno et al., 2010; Salcedo et al., 2007; Zuellig et al., 2014). For example, in avian primary sensory and motor neurons *in vitro*, treatment with DSp was found to cause a dose-dependent decrease in neurite branching, indicative of neuronal toxicity (Penno et al., 2010). Additional toxic effects of abnormal sphingoid bases were also reported in these chick neurons, including reduced neurite outgrowth, retraction of existing neurites and disturbed actin-neurofilament interactions (Penno et al., 2010).

Although several lines of evidence now show that exposure to these abnormal deoxsphingoid bases causes morphological changes, the cellular pathomechanisms that underlie these toxic effects remain unresolved. Previous studies have examined the molecular targets of deoxysphingoid bases primarily in cell lines (Alecu et al., 2016b; Cuadros et al., 2000; Gable et al., 2010; Salcedo et al., 2007; Sayano et al., 2016). Exogenous application of DSp to cell lines resulted in an upregulation of the ER stress markers GADD153 (Gable et al., 2010) and spliced X-box binding protein 1 (XBP1, Alecu et al., 2016b). In addition to showing that exogenous DSp treatment causes ER stress, Alecu et al. (2016b) also showed that DSp application causes both morphological and functional mitochondrial changes. Findings in HSN-1 patient lymphoblasts further suggest that mitochondrial abnormalities may play a role in HSN-1 pathology (Myers et al., 2014; Stimpson et al., 2015).

However, fewer studies have examined the effects of deoxysphingoid base targets in neuronal cells (Alecu et al., 2016b; Guntert et al., 2016). Alecu et al. (2016b) demonstrated that exogenous application of DSp to primary DRG cells resulted in mitochondrial swelling. Moreover, studies in mammalian cortical neurons suggest there may be an interaction between DSp and *N*-methyl-d-aspartate (NMDA) receptor signalling (Guntert et al., 2016).

In this study, we set out to examine whether exogenous deoxy-SPT products are neurotoxic to primary mammalian neurons by measuring parameters indicative of cell viability, including neurite outgrowth and cell survival. Moreover, we also explore the potential pathomechanisms underlying such neurotoxicity by examining intracellular Ca^2+^ handling and mitochondrial function in both mammalian primary motor and sensory neurons *in vitro*.

## 2 Materials and methods

Animals were sacrificed by Schedule 1 methods as outlined in the UK Animals (Scientific Procedures) Act (1986), and approved by the UCL Institute of Neurology Animal Welfare Ethical Review Board. All reagents were obtained from ThermoFisher Scientific unless otherwise specified.

### 2.1 Primary mixed motor neuron cultures

For primary mixed motor neuron (MN) cultures, the ventral spinal cord was dissected from embryonic day 12.5–13.5 (E12.5-E13.5) mouse embryos (C57BL/6 J, Charles River Laboratories). Ventral horns were digested in trypsin (0.025%) for 10 mins at 37 °C. The tissue was then triturated in DNAse I (0.1 mg/ml, Sigma) in 0.4% bovine serum albumin (BSA) in Leibovitz's L15 medium. Supernatant was collected and the remaining pellet triturated a second time in DNAse I (0.02 mg/ml) in 0.4% BSA in Leibovitz's L15 medium. Supernatants from both trituration steps were combined and centrifuged through a BSA cushion (4% in Leibovitz's L15 medium) at 380 ×*g* for 5 mins to pellet cells. The supernatant was discarded and the cell pellet re-suspended in supplemented neurobasal medium containing 2% B27 supplement, 0.5 mM GlutaMAX, 25 μM 2-mercaptoethanol, 2% horse serum, 0.1 ng/ml murine glial cell line-derived neurotrophic factor (GDNF, Peprotech), 0.5 ng/ml human or rat ciliary neurotrophic factor (CNTF by Peprotech), 0.1 ng/ml human brain-derived neurotrophic factor (BDNF, Peprotech) and penicillin/streptomycin (50 units per ml/50 μg per ml). Cells were plated onto 24-well plates with glass coverslips at a cell density of 5 × 10^4^ cells/well for cell survival and neurite outgrowth assays. For live-cell imaging, cells were plated onto glass bottom dishes (35 mm petri dish with 14 mm microwell, MatTek) at a cell density of 1 × 10^5^ cells/well or 8 well μ-slides (Ibidi) at a cell density of 3 × 10^4^ cells/well. All culture dishes were pre-coated overnight with poly-L-ornithine (3 μg/ml, Sigma) followed by laminin (5 μg/ml, Sigma) for 2 h, prior to seeding cells. These mixed ventral horn cultures were maintained in supplemented neurobasal medium (as above), under standard culture conditions (37 °C, 5% CO_2_). MNs were considered immature after 1 day *in vitro* (DIV) and mature after 5 or more DIV, due to their general morphology as well as the expression of key MN characteristics, such as expression of glutamate receptors (Van Den Van Den Bosch et al., 2000).

### 2.2 Primary dorsal root ganglia cultures

For primary dorsal root ganglia (DRG) cultures, DRG were harvested from 3 to 8 day old mice (C57BL/6). Spinal columns were removed, the DRG exposed and dissected into sterile HBSS (Ca^2+^ and Mg^2+^ free) supplemented with penicillin/streptomycin (100 units per ml/100 μg per ml). Dissected ganglia were then incubated in pre-warmed HBSS (Ca^2+^ and Mg^2+^ free) containing 60 units of papain (Sigma) at 37 °C for 10 min with gentle rotation. Papain solution was then removed and ganglia re-suspended in pre-warmed HBSS containing collagenase type II (0.1%) and dispase (1.5 units/ml) at 37 °C for 45 min with gentle rotation. DRG were then gently triturated in order to dissociate them into a cell suspension. Cells were plated onto glass bottom dishes (35 mm petri dish with 14 mm microwell) or 8 well μ-slides (Ibidi) pre-coated with poly-d-lysine (100 μg/ml, Sigma) and laminin (5 μg/ml). Mixed DRG cultures were cultured in neurobasal medium supplemented with B27 supplement (2%), fetal calf serum (1%), 2 mM GlutaMAX, mouse nerve growth factor (50 ng/ml, Promega) and penicillin/streptomycin (50 units per ml/50 μg per ml). DRG cells were incubated under standard culture conditions (37 °C, 5% CO_2_) and used for experiments after 3–5 DIV.

### 2.3 Drug treatments

Metabolites of SPT enzyme products were purchased from Avanti Polar Lipids and dissolved in ethanol as stock solutions (1 mM). Cells at different stages after plating were either left untreated or treated with sphinganine (Sp), deoxysphinganine (DSp), deoxymethylsphinganine (DMSp) or ethanol (as a vehicle control, 0.1%), at final concentrations ranging from 0.1 μM to 10 μM. The duration of treatment ranged from 5 min to 6 days, as described in Results. In some experiments, cultures were co-treated with 1 μM cyclosporine A (CsA, Sigma), a mitochondrial permeability transition pore (mPTP) blocker.

#### 2.3.1 Immunocytochemistry

For cell survival and neurite outgrowth assays, at the end of each treatment regime, the cells were fixed in 4% paraformaldehyde for 20 min then washed in phosphate buffered saline (PBS). Prior to primary antibody labelling, cells were permeabilised in PBS containing 0.1% Triton X-100 (Sigma) and blocked in 5% goat serum. Mouse anti-β-III tubulin (BioLegend 801201) was used as a pan-neuronal marker (1:1000), followed by Alexa Fluor® 488 labelled anti-mouse IgG (H + L) secondary antibody (1:1000, raised in goat). DAPI (1:2000, Sigma) was used to stain nucleic acids (nuclei).

#### 2.3.2 Cell survival and neurite outgrowth assays

Cultures were immunostained for the neuronal marker anti-β-III tubulin and stained with the nuclear marker DAPI. The cells were then imaged using the 20× objective of a Leica epifluorescence light microscope and the Leica Application Suite software. Images were taken for cell and neurite counts, as well as assessment of neurite length. A minimum of five representative images measuring 655 × 491 μm were taken per condition, per experiment. Cells positive for β-III tubulin and DAPI counter-stain were counted as neurons to establish the total number of cells surviving. Neurite outgrowth was assessed by tracing from the cell body to neurite tip. When branched, the longest neurite was measured and the shorter branch counted as a separate neurite.

#### 2.3.3 Intracellular Ca^2+^ measurements

Cultures were washed in recording medium (156 mM NaCl, 10 mM HEPES, 10 mM d-glucose, 3 mM KCl, 2 mM MgSO_4_, 2 mM CaCl_2_, 1.25 mM KH_2_PO_4_, pH 7.35) and then loaded with 5 μM fura-2 AM (Molecular Probes by Life Technologies) in the above recording medium supplemented with pluronic F127 (0.04%), for 30 min, at room temperature. Fura-2 was then replaced with recording medium and dye accumulation optically detected by charge-coupled device (CCD) camera microscopy following excitation at wavelengths 340 nm and 380 nm using the computer programme Andor iQ 1.9 Imaging. The ratio of fura-2 emission at 510 nm following excitation at 340/380 nm wavelengths was used as a readout measure of intracellular Ca^2+^ concentration; thus, an increase in this ratio signifies an increase in free Ca^2+^ present. Relative ER Ca^2+^ concentration and mitochondrial Ca^2+^ concentration were measured in Ca^2+^-free recording medium containing 0.5 mM EGTA, first using thapsigargin (10 μM, Sigma) to estimate ER Ca^2+^ (Michelangeli and East, 2011), followed by ionomycin treatment (10 μM, Sigma), to estimate mitochondrial Ca^2+^ (Abramov and Duchen, 2003). Under these conditions ionomycin application results in the release of Ca^2+^ from intracellular Ca^2+^ stores, including mitochondria. Other cellular compartments also release Ca^2+^ following ionomycin application but the greatest contribution is likely to come from mitochondria (Abramov and Duchen, 2003). Membrane depolarization was achieved using a high potassium-containing (135 mM KCl) recording medium. Store-operated Ca^2+^ channel entry was measured using thapsigargin (10 μM) in Ca^2+^-free recording medium, followed by replacement with standard, Ca^2+^-containing recording medium. In some experiments, 1 μM carbonyl cyanide-*p*-trifluoromethoxyphenylhydrazone (FCCP, Sigma) was introduced 2 mins prior to thapsigargin treatment and included in recording media throughout the experiment in order to render mitochondria non-functional and to test the contribution of mitochondrial Ca^2+^ buffering to the overall intracellular Ca^2+^ increase. Relative ER Ca^2+^, mitochondrial Ca^2+^, membrane-depolarization induced Ca^2+^ influx and store-operated Ca^2+^ entry were all calculated by subtracting the baseline fura-2 ratio from the relevant peak amplitude fura-2 concentration. The drugs and conditions used to manipulate intracellular Ca^2+^ handling are summarised in Table 1. In all experiments, dying or dead cells not responding to treatment were excluded from the analysis.

**Table 1:** Drugs used to explore intracellular Ca^2+^ handling: The Table summarises the treatments used to measure physiological Ca^2+^ handling parameters.

Drug	Target of drug	Experimental condition	Physiological measurement
Thapsigargin	SERCA pump inhibitor	Ca^2+^-free recording medium	ER [Ca^2+^]
		Apply in Ca^2+^-free recording medium and then re-introduce Ca^2+^ to the external medium	SOC channel entry
Ionomycin	Non-selective ionophore	Apply after thapsigargin, in Ca^2+^-free recording medium	[Ca^2+^] in mitochondrial and other intracellular stores
135 mM [K^+^]	Plasma membrane depolarization	Normal recording medium	Membrane-depolarization induced Ca^2+^ influx
FCCP	Protonophore to abolish mitochondrial membrane potential	Apply FCCP throughout the experiment	To determine mitochondrial contribution

#### 2.3.4 Measurement of mitochondrial membrane potential (∆ψ_m_) and total mitochondrial area

Primary cultures at 5–8 days *in vitro* (DIV) (MNs) or 3–5 DIV (DRG neurons) were treated with the sphingoid bases for 2 h prior to live cell imaging. Cultures were loaded with tetramethylrhodamine methyl ester (TMRM) (20 nM) in recording medium (as described above) supplemented with pluronic F127 (0.04%), for 30 min, at room temperature before imaging (no washes were performed prior to imaging). Immediately before imaging, cells were additionally loaded with the live-cell labelling dye calcein blue, AM (1 μM). *Z*-stack images were taken on Zeiss Laser Scanning 710 (MNs) or 780 (DRG neurons) confocal microscopes using the lasers 405 nm and 561 nm and the ZEN LE Digital Imaging 2009 or 2012 software. Z-stacks (minimum 720 × 720 pixels and 12 bit depth) were compressed to maximal intensity per pixel and thresholded (separately to select for either TMRM signal solely from mitochondria or calcein blue signal) using ImageJ version 1.51n. In processed images calcein blue was used to outline individual cells. TMRM intensity (as a readout measure for ∆ψ_m_) was calculated from the cell soma and expressed as a percentage relative to the mean TMRM intensity in control cells, for each independent imaging session. Total mitochondrial area (determined using TMRM) was expressed as a percentage of the cell soma size (determined using calcein blue), from thresholded images as described above.

#### 2.3.5 Statistical analysis

Statistical analyses were performed using GraphPad Prism Version 6.0. Data were assumed to be normally distributed unless evidence was provided otherwise by the D'Agostino-Pearson normality test. Thus, statistical significance was determined by one- or two-way ANOVA with Dunnett's multiple comparison tests, Kruskal-Wallis with Dunn's multiple comparison tests, unpaired *t*-test or Mann-Whitney test, as appropriate. * *P* < 0.05, ** *P* < 0.01 and *** *P* < 0.001.

## 3 Results

To determine whether abnormal deoxysphingoid bases are neurotoxic to mammalian motor neurons, which together with sensory neurons are typically affected in HSN-1, primary motor neuron cultures (MNs) were treated with either the normal enzymatic product sphinganine (Sp), the atypical products, deoxysphinganine (DSp) and deoxymethylsphinganine (DMSp), or a vehicle control (ethanol), at a range of concentrations and for varying treatment durations. The concentrations chosen were based on deoxysphingoid base concentrations previously measured in HSN-1 patient plasma (Auer-Grumbach et al., 2013; Murphy et al., 2013; Penno et al., 2010; Rotthier et al., 2011; Suriyanarayanan et al., 2016).

### 3.1 Deoxysphingoid bases are toxic to mammalian motor neurons

**Fig. 1:**
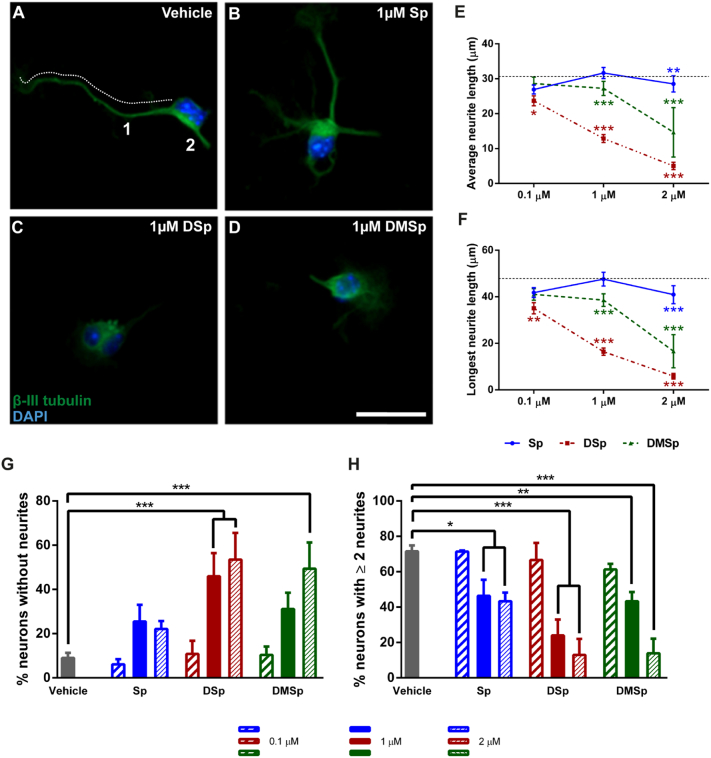
Treatment with deoxysphingoid bases reduce neurite outgrowth in primary motor neurons, in a dose-dependent manner. (A-D) Primary MNs were grown for 24 h and then treated with either vehicle (ethanol) or with sphingoid bases. The number of neurites per neuron was counted as indicated and neurite length was measured by tracing neurites, as illustrated by the dotted line in (A). The cells were stained for DAPI (blue) and immunostained for β-III tubulin (green). Scale bar = 25 μm. Treatment with the deoxysphingoid bases reduces (E) the average neurite length, and (F) the average length of the longest neurite, in a dose-dependent manner. In E and F the black dotted line indicates the average or average longest neurite length of MNs treated with an ethanol vehicle control. These data are not normally distributed and thus displayed statistics indicate comparisons to vehicle control using Kruskal-Wallis (*P* < 0.001) and Dunn's multiple comparisons tests. Two-way ANOVA was also performed on the average neurite length (*P* < 0.001, concentration, *P* < 0.001, treatment, *P* < 0.001, interaction) and longest neurite length (*P* < 0.001, concentration, *P* < 0.001, treatment, *P* < 0.001, interaction). (G) The percentage of MNs with no neurite outgrowth following treatment with the sphingoid bases at a range of concentrations. The percentages of neurons with no neurite outgrowth were compared to vehicle controls using two-way ANOVA and Dunnett's multiple comparison test: *P* < 0.001, concentration, *P* < 0.001, treatment, *P* < 0.05, interaction. (H) The percentage of MNs with 2 or more neurites following treatment with the sphingoid bases at a range of concentrations. The percentages of neurons with no neurite outgrowth were compared to vehicle controls using two-way ANOVA and Dunnett's multiple comparison test: *P* < 0.001, concentration, *P* < 0.001, treatment, *P* < 0.001, interaction. Error bars represent S.E.M. *P* values: * < 0.05; ** < 0.01; *** < 0.001. n = 18–383 cells per condition, from 3 to 5 independent experiments.

Previous studies have shown that atypical sphingoid bases have deleterious effects on immature primary chick MNs when treated within the first 24 h of plating, causing decreased neurite outgrowth (Penno et al., 2010). Therefore, in this study we first examined the effects of 24 h sphingoid base treatments on neurite outgrowth in immature mouse MNs at 1 day *in vitro* (DIV) by immunostaining for the neuronal marker β-III tubulin. Fig. 1A–D shows representative examples of mouse MNs treated with vehicle, or 1 μM Sp, DSp or DMSp for 24 h; the measurement criteria used for neurite outgrowth assays are illustrated in Panel A. Compared to untreated neurons, ethanol treatment alone caused an increase in average and longest neurite lengths (Supplementary Fig. S2). Therefore, in all subsequent experiments the effects of sphingoid base treatments on neurite outgrowth were compared to vehicle controls.

### 3.2 Deoxysphingoid bases reduce neurite outgrowth in motor neurons

The average neurite length was decreased in MNs treated with Sp, DSp and DMSp, and this effect was exacerbated by increasing concentrations (Fig. 1E). Indeed, DSp treatment at a concentration as low as 0.1 μM, significantly decreased average neurite length, from 30.4 ± 1.3 μm in vehicle controls (indicated by the dotted line in Fig. 1E) to 23.7 ± 1.4 μm in DSp-treated cells (*P* < 0.05). Although no significant decrease in MN neurite length was observed with 0.1 μM DMSp treatment, cells treated with 1 μM DMSp had a reduced neurite length of 27.2 ± 2.0 μm (*P* < 0.001). In 1 μM DSp-treated cells MN neurite length was further decreased to 12.9 ± 1.1 μm (*P* < 0.001). Interestingly, treatment with the typical enzyme product, Sp, was also moderately toxic, with 2 μM treatment causing a small but significant decrease (to 28.5 ± 2.3 μm) in average MN neurite length (*P* < 0.01). A similar pattern of toxicity was observed when the longest neurite length per MN was examined (Fig. 1F).

The number of neurites per MN was also assessed, as an indication of neuronal maturation (Fig. 1G–H). Following treatment with increasing concentrations (0.1–2 μM) of DSp or DMSp, there was an increase in the number of neurons with no neurites (Fig. 1G) and a decrease in the number of neurons with two or more neurites (Fig. 1H), indicating that deoxysphingoid base treatments have detrimental effects on neuronal complexity. Following treatment with 1 μM DSp, 46 ± 10% of MNs had no neurites and 24 ± 9% of MNs had two or more neurites. This contrasts to vehicle treated cultures, in which only 9 ± 2% of MNs had no neurites and the majority of MNs, 72 ± 3%, had a more complex dendritic arborisation pattern, with at least two or more neurites.

In summary, treatment with DSp and DMSp causes a rapid and dose-dependent reduction in neurite outgrowth in primary MNs *in vitro* (Fig. 1A–D), manifesting both as a decrease in neurite length (Fig. 1E–F) and a decrease in the number of neurite projections (Fig. 1G–H) when compared to vehicle treated controls.

### 3.3 Deoxysphingoid bases cause motor neuron death

The effect of increasing concentrations of sphingoid bases on MN survival was also determined. In these experiments, the number of β-III tubulin^+^ cells in each culture was counted and expressed as a percentage of MN survival in control cultures. Previous studies have shown that treatment of immature (<1 DIV) chick neurons (DRG neurons and MNs) with deoxysphingoid bases for a short period (24 h) results in neuronal toxicity, as determined by assessment of neurite outgrowth (Penno et al., 2010); however, this study did not assess the effect on neuronal survival. In the present study, we therefore first established whether a similar treatment regime was sufficiently toxic to mammalian MNs to affect neuronal survival. We also examined the effects of longer treatment durations, as well as the effects on more mature (5 DIV) MNs.

**Fig. 2:**
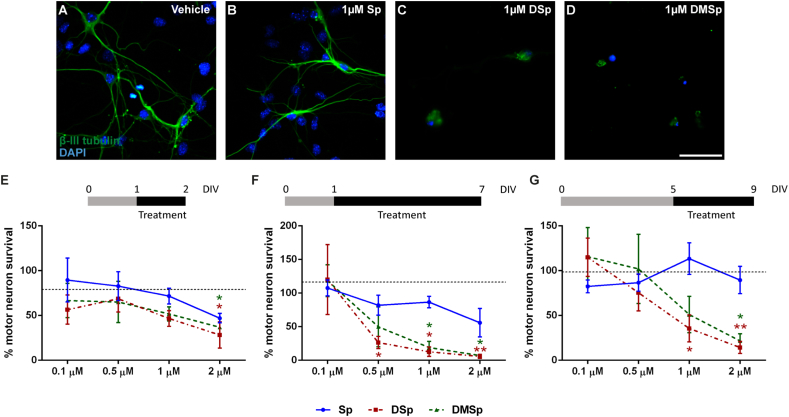
Treatments with deoxysphingoid bases reduce survival of primary motor neurons, in a dose-dependent manner. (A-D) Representative images of primary MNs grown for 24 h before being treated with either (A) vehicle (ethanol) control, (B) Sp, (C) DSp or (D) DMSp. MNs were fixed and stained with DAPI (blue) and immunostained for β-III tubulin (green) 6 days following treatment, at 7 DIV. Scale bar = 50 μm. (E) MN survival following treatment at 1 DIV for 24 h, with different doses of sphingoid bases, expressed as a percentage relative to control. MN survival was compared to survival in vehicle treated cultures using two-way ANOVA and Dunnett's multiple comparison test: *P* < 0.05, concentration, *P* < 0.01, treatment, *P* = 0.792, interaction. (F) MN survival following treatment at 1 DIV for 6 days, with different doses of sphingoid bases, expressed as a percentage relative to the untreated control. MN survival was compared to survival in vehicle treated cultures using two-way ANOVA and Dunnett's multiple comparison test: *P* < 0.01, concentration, *P* < 0.001, treatment, *P* = 0.393, interaction. (G) MN survival following treatment at 5 DIV for 4 days, with different doses of sphingoid bases, expressed as a percentage relative to the untreated control. MN survival was compared to survival in vehicle treated cultures using two-way ANOVA and Dunnett's multiple comparison test: *P* < 0.01, concentration, *P* < 0.05, treatment, *P* < 0.05, interaction. The black dotted lines in E-G represent MN survival in vehicle treated cultures. Error bars represent S.E.M. *P* values: * < 0.05; ** < 0.01. n = 4–6 independent experiments per condition.

At 1 DIV, MN cultures were treated for 24 h with increasing concentrations of Sp, DSp and DMSp (Fig. 2E). Following 24 h treatment with 2 μM DSp and DMSp, MN survival was reduced to 28 ± 15% (*P* < 0.05) and 37 ± 10% (*P* < 0.05), respectively.

As shown in the representative images in Fig. 2A–D, longer term treatment of immature MNs (at 1 DIV) with deoxysphingoid bases resulted in a dramatic loss of MNs. Notably, in this long-term, 6 day treatment paradigm (indicated in the schematic in the upper panel of Fig. 2F), the significant loss of MNs was also accompanied by death of non-neuronal cells such as fibroblasts, so that few DAPI^+^, β-III tubulin^−^ cells were observed. The extent of MN survival was quantified, and a dose-dependent decrease in MN survival was observed in response to DSp and DMSp treatments (Fig. 2F). In cultures treated with 1 μM DSp or DMSp, only 13 ± 7% (*P* < 0.05) and 19 ± 9% (*P* < 0.05) of MNs survived, respectively.

Since MNs at 1 DIV are relatively immature, the effect of sphingoid base treatments on more mature MN cultures was also examined, by treating MNs at 5 DIV for 4 days (shown in the schematic in the upper panel of Fig. 2G). This was the longest treatment period possible in primary MN cultures, since even untreated MNs begin to deteriorate in culture past 10–14 DIV. These more mature MNs also displayed susceptibility to deoxysphingoid base toxicity and in this treatment paradigm, only 36 ± 15% (*P* < 0.05) and 51 ± 20% (*P* < 0.05) of MNs survived following 1 μM DSp and DMSp treatments, respectively.

These results show that the abnormal enzyme products DSp and DMSp, cause a dose-dependent toxicity in immature and mature cultured primary MNs.

### 3.4 Deoxysphingoid bases cause abnormal Ca^2+^ handling in motor and sensory neurons

Having established that deoxysphingoid base treatments are toxic to mammalian neurons, we next examined the potential mechanisms underlying the potent neurotoxicity of these abnormal enzyme products. Since immature MNs (1 DIV) die rapidly upon exposure to deoxysphingoid bases, within 24 h (Fig. 2E), it is difficult to dissect the mechanisms underlying cytotoxicity in these cells using this experimental protocol. Therefore, in the following experiments, designed to examine the underlying pathomechanisms of deoxysphingoid base neurotoxicity, only more mature MNs (≥5 DIV) were examined. Moreover, mature MNs do not show the increased vulnerability to the normal enzyme product, Sp, observed in immature MNs (Fig. 2).

### 3.5 Deoxysphingoid bases have no effect on resting cytosolic Ca^2+^

SPT is an ER membrane protein and it is well known that the ER plays a major role in cellular Ca^2+^ homeostasis. Since changes in intracellular Ca^2+^ levels are an important early indicator of cell stress (Mattson, 2000; Trump and Berezesky, 1995), we used the ratiometric dye fura-2 to examine intracellular Ca^2+^ in neurons treated with the deoxysphingoid bases.

Mature MNs (≥5 DIV) were treated acutely for 2 h with 1 μM of sphingoid bases, and the effects on baseline cytosolic Ca^2+^ levels were determined. Following 2 h treatment with either DSp or DMSp there was no significant difference in resting cytosolic Ca^2+^ levels (Supplementary Fig. S3A; fura-2 ratio: 0.77 ± 0.01 in DSp treated cells and 0.73 ± 0.01 in DMSp treated cells compared to vehicle-treated cells (ratio: 0.77 ± 0.01)), indicating that short-term treatment with deoxysphingoid bases does not alter resting cytosolic Ca^2+^ homeostasis and these MNs appear largely healthy.

### 3.6 Deoxysphingoid bases cause rapid depletion of ER Ca^2+^ in motor and sensory neurons

**Fig. 3:**
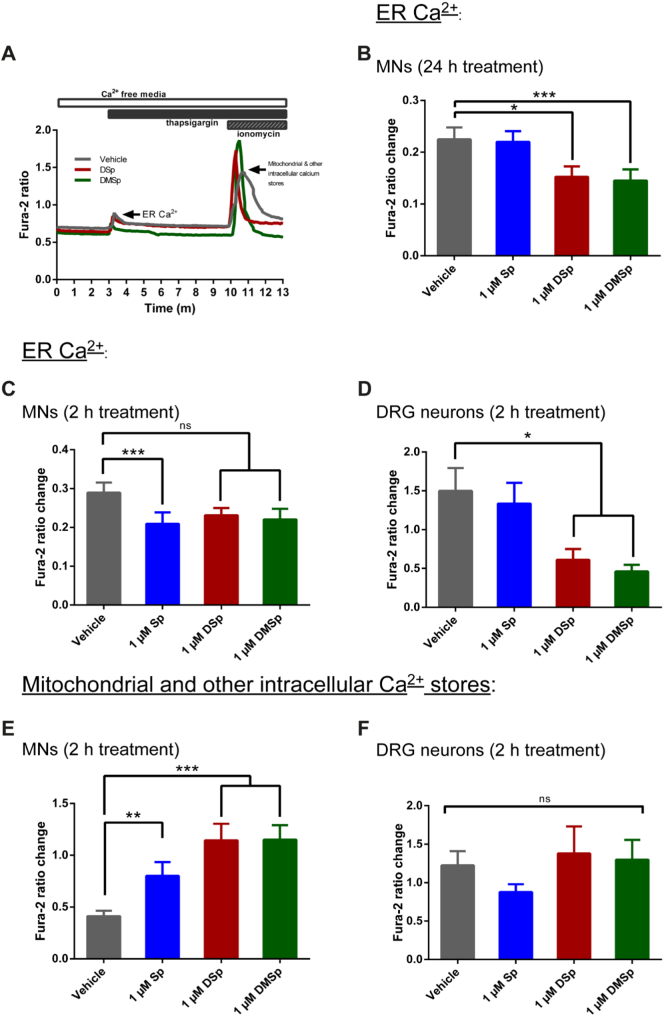
Treatments with deoxysphingoid bases cause depletion of ER Ca^2+^ and mitochondrial Ca^2+^ loading in neurons. (A) A typical trace (from a MN culture) indicating how thapsigargin and ionomycin were used to estimate ER and mitochondrial Ca^2+^ levels. (B) At 5–8 DIV, MNs were treated with either vehicle control (ethanol) or the sphingoid bases for 24 h prior to live cell imaging. Thapsigargin was used to estimate ER Ca^2+^. Average ER Ca^2+^ was established from 42 to 55 cells per condition, from 4 to 5 independent experiments. (*P* < 0.001, Kruskal-Wallis). (C) At 5–8 DIV, MNs were treated with either vehicle (ethanol) or the sphingoid bases for 2 h prior to live cell imaging, and as above, thapsigargin used to estimate the ER Ca^2+^. Average ER Ca^2+^ was established from 94 to 184 cells, from 11 to 16 independent experiments. (*P* = 0.003, Kruskal-Wallis). (D) At 3–5 DIV, DRG neurons were treated for 2 h with either vehicle control (ethanol) or the sphingoid bases for 24 h prior to live cell imaging. Thapsigargin was used to estimate ER Ca^2+^ and average ER Ca^2+^ was established from 28 to 38 cells, from 3 to 4 independent experiments. (*P* < 0.001, Kruskal-Wallis). (E) At 5–8 DIV, MNs were treated for 2 h prior to live cell imaging. Thapsigargin and ionomycin were used to estimate mitochondrial Ca^2+^. Average mitochondrial Ca^2+^ was established from 36 to 44 cells per condition, from 4 to 5 independent experiments. (*P* < 0.001, Kruskal-Wallis). (F) At 3–5 DIV, DRG cultures were treated with either vehicle control (ethanol) or the sphingoid bases for 2 h prior to live cell imaging. Mitochondrial Ca^2+^ was estimated as above, and average mitochondrial Ca^2+^ was established from 32 to 41 cells per condition, from 4 independent experiments. (*P* = 0.063, Kruskal-Wallis). Error bars represent S.E.M. For statistical comparison, each treatment group was compared to vehicle control using Kruskal-Wallis and Dunn's multiple comparisons tests. *P* values: * < 0.05; ** < 0.01; *** < 0.001.

Although resting cytosolic Ca^2+^ levels were not altered by acute exposure to deoxysphingoid bases, it is possible that this is the result of effective Ca^2+^ buffering by intracellular organelles, such as the ER and mitochondria. We therefore examined the effects of deoxysphingoid base treatments on ER Ca^2+^. Mature MNs (≥ 5 DIV) were first treated for 24 h with 1 μM deoxysphingoid bases and relative ER Ca^2+^ levels determined by measuring the change in cytosolic Ca^2+^ following treatment with the sarco/endoplasmic reticulum Ca^2+^-ATPase (SERCA) pump inhibitor, thapsigargin, which causes the ER to release Ca^2+^ into the cytosol. As these experiments were performed in the absence of extracellular Ca^2+^ the resulting change in cytosolic Ca^2+^ can be used to infer a relative ER Ca^2+^, as shown in a typical Ca^2+^ recording experiment shown in Fig. 3A. Treatment of MNs with deoxysphingoid bases caused a significant reduction in the relative ER Ca^2+^ in MNs. Thus, in DSp treated MNs the fura-2 ratio for relative ER Ca^2+^ was reduced to 0.15 ± 0.02 compared to 0.22 ± 0.02 in the vehicle treated MNs (*P* < 0.05), indicative of ER stress (Fig. 3B). In order to determine if ER stress is an early event in deoxysphingoid base-mediated neurotoxicity, we shortened the treatment paradigm to just 2 h. Intriguingly, 2 h treatment with the typical enzyme product, Sp, caused a significant decrease in the relative ER Ca^2+^ levels, whereas the deoxysphingoid bases caused only non-significant reductions in the relative ER Ca^2+^ to 0.23 ± 0.02 and 0.22 ± 0.03 in DSp and DMSp treated cells, respectively, in comparison to 0.29 ± 0.03 in vehicle treated cells (Fig. 3C). Notably, treatment with deoxysphingoid bases over this 2 h time frame did not cause any change in SPTLC1 protein expression levels (Supplementary Fig. S4).

We next investigated whether treatment with deoxysphingoid bases also induces ER stress in primary sensory DRG cells. In DRG cultures treated for 2 h with deoxysphingoid bases, we observed a dramatic reduction in the relative ER Ca^2+^ compared to vehicle-treated DRG neurons (Fig. 3D); DRG neurons treated with 1 μM DSp or DMSp displayed a relative ER fura-2 ratio of 0.61 ± 0.14 and 0.46 ± 0.09, respectively, in contrast to vehicle-treated DRG neurons, which had a relative ER fura-2 ratio of 1.50 ± 0.29.

A comparison of the results from MNs and DRG neurons suggests that deoxysphingoid bases may induce ER stress more readily in DRG neurons than in MNs, with deoxysphingoid base-induced ER stress evident in DRG neurons after 2 h treatment, yet similar depletion of relative ER Ca^2+^ in MNs was observed only after 24 h treatment. However, there was a clear difference between the relative ER Ca^2+^ levels measured in these two cell types, so that the relative ER Ca^2+^ was substantially higher in DRG neurons (1.50 ± 0.29, Fig. 3D) than in MNs (0.29 ± 0.03, Fig. 3C), which may allow us to detect changes in Ca^2+^ more readily than in MNs. Despite this, a direct comparison of ER Ca^2+^ levels in the two populations of neurons may not be appropriate as these neurons have a number of key differences, for example their size, excitability and indeed Ca^2+^ buffering capacity.

### 3.7 Deoxysphingoid bases cause mitochondrial Ca^2+^ loading in motor neurons

Since mitochondria also play an important role in the regulation of intracellular Ca^2+^ in neurons, mitochondrial Ca^2+^ levels were also examined. Mitochondrial Ca^2+^ was estimated using thapsigargin and the ionophore, ionomycin, in the absence of extracellular Ca^2+^ (as indicated in the experiment shown in Fig. 3A). This technique allows an estimation of mitochondrial Ca^2+^ to be made (Abramov and Duchen, 2003; Hoek et al., 1995). However, it should be noted that although the ER contribution to the ionomycin-induced peak is largely eliminated due to advanced emptying of the ER Ca^2+^ stores with thapsigargin, it is possible that intracellular organelles distinct from the ER and the mitochondria may also provide a minor contribution of Ca^2+^. Following 2 h treatment of MNs with the sphingoid bases, there was a significant elevation in mitochondrial Ca^2+^ (Fig. 3E). In vehicle treated MNs, the mitochondrial fura-2 ratio was 0.41 ± 0.05, but this ratio more than doubled in DSp-and DMSp-treated MNs to 1.14 ± 0.16 (*P* < 0.001) and 1.15 ± 0.14 (*P* < 0.001), respectively. This shift towards higher mitochondrial Ca^2+^ was also observed, to some extent, in Sp-treated MNs, which had an average fura-2 ratio of 0.80 ± 0.13 (*P* < 0.01).

In contrast, in DRG neurons, the same 2 h treatment regime had no effect on mitochondrial Ca^2+^ levels (Fig. 3F), so that in DSp-treated DRG neurons, the average mitochondrial Ca^2+^ concentration was 1.38 ± 0.35 compared to 1.23 ± 0.19 in vehicle-treated DRG neurons. However, as observed with ER Ca^2+^ levels, mitochondrial Ca^2+^ levels in control DRG cells were substantially higher than in control MNs (compare Fig. 3E and F).

### 3.8 Cell membrane depolarization-induced Ca^2+^ influx is unaffected in deoxysphingoid base-treated motor neurons

**Fig. 4:**
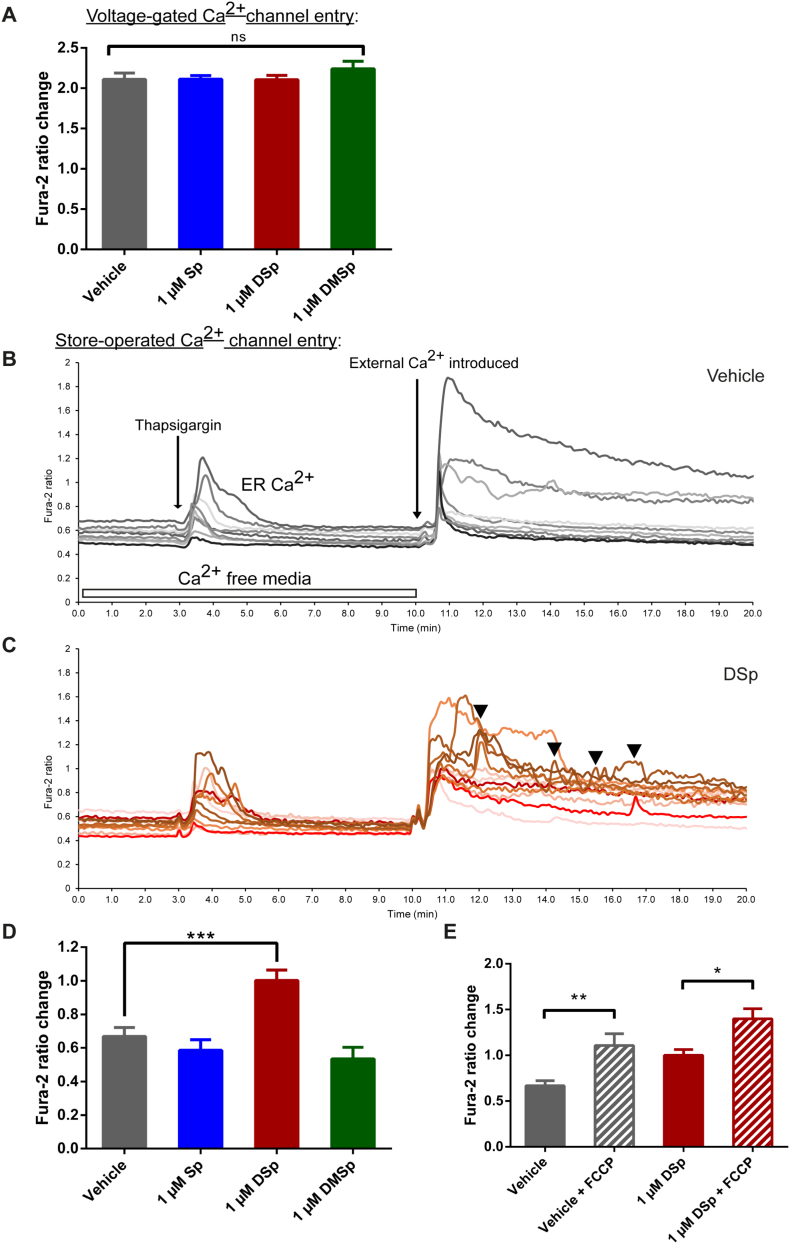
Deoxysphingaine causes dysregulation of store-operated Ca^2+^ (SOC) channels. (A-E) At 5–8 DIV, MNs were treated with vehicle control or sphingoid bases 2 h prior to live cell imaging with fura-2. (A) High potassium was used to depolarize the plasma membrane potential and trigger opening of voltage-gated Ca^2+^ channels. Average membrane depolarization-induced Ca^2+^ influx per cell was calculated from 23 to 52 cells per condition, from 2 to 3 independent experiments. (*P* = 0.743, one-way ANOVA). (B-E) SOC channel entry was measured using thapsigargin and subsequent introduction of Ca^2+^ to the external recording medium. (B-C) Example traces showing SOC channel entry in vehicle (B) and DSp (C) treated cells. Each differentially shaded line represents a different cell. Arrowheads in Fig. 4C indicate subsequent influxes of Ca^2+^ following the initial SOC channel peak influx in the DSp-treated MNs. (D) Average Ca^2+^ entry through SOC channels was calculated from 36 to 97 cells per condition, from 5 to 9 independent experiments. (*P* < 0.001, Kruskal-Wallis). (E) SOC channel entry was measured with and without co-treatment with FCCP. The average SOC channel Ca^2+^ influx was established from 38 to 97 cells per condition, from 4 to 9 independent experiments. Error bars represent S.E.M. For statistical comparison, each treatment group was compared to vehicle control, unless otherwise indicated. Data following Gaussian distribution was compared to vehicle control using One-way ANOVA and Dunnett's multiple comparisons tests. Non-normally distrusted data was compared to vehicle control using Kruskal-Wallis and Dunn's multiple comparisons tests. Pairwise comparisons were made using Mann-Whitney tests. *P* values: * < 0.05; ** < 0.01; *** < 0.001.

We next investigated the source of the increased mitochondrial Ca^2+^ observed following acute, 2 h treatment of MNs with the sphingoid bases. Firstly, we considered whether the elevated Ca^2+^ levels might be a consequence of changes at the cell membrane. We therefore investigated the effect of deoxysphingoid base treatments on membrane depolarization-induced Ca^2+^ influx, such as those through voltage-gated Ca^2+^ channels. In these experiments, potassium was used to depolarize the plasma membrane, thus triggering the opening of voltage-gated Ca^2+^ channels. As can be seen in Fig. 4A, we observed no difference in the fura-2 ratios representing membrane depolarization-induced Ca^2+^ influx, between the vehicle control (2.11 ± 0.08), and any of the sphingoid base treatments; Sp (2.11 ± 0.05), DSp (2.11 ± 0.05), DMSp (2.24 ± 0.10).

### 3.9 DSp causes increased store-operated Ca^2+^ (SOC) channel entry

Next, we examined whether the observed increase in mitochondrial Ca^2+^ might originate from a second class of cell membrane channels, by examining entry of Ca^2+^ through the ER-regulated store-operated Ca^2+^ (SOC) channels. These plasma membrane channels are regulated by intracellular Ca^2+^ levels, namely the ER (Putney et al., 2016). To investigate SOC channel entry, thapsigargin was used to empty Ca^2+^ from the ER, which in turn results in the opening of SOC channels on the cell membrane, in a bid to replenish the ER Ca^2+^ stores, as shown in experiments depicted in Fig. 4B and C. Thus, when the external Ca^2+^-free medium was replaced with Ca^2+^-containing medium, the influx of Ca^2+^ through SOC channels was measured. As can be seen in Fig. 4D, treatment of MNs with DSp for 2 h resulted in an increase in Ca^2+^ influx through SOC channels compared to vehicle control treated MNs, with a fura-2 ratio of 1.00 ± 0.06 and 0.67 ± 0.05 respectively (*P* < 0.001). Frequently, we also observed fluctuation in fura-2 ratios in DSp-treated MNs following Ca^2+^ influx through SOC channels when compared to vehicle treated MNs (Fig. 4C). This may be indicative of dysregulation of these channels or other disruption at the cell membrane and, indeed, biochemical studies of the properties of deoxysphingoid bases and their derivatives propose that these compounds may have the ability to affect normal cell membrane function (Jimenez-Rojo et al., 2014). It is unlikely that the abnormal sphingolipids have an acute effect on SOC channels directly, since acute, 5 min treatment does not cause any change in cytosolic fura-2 ratios in these cells, indicating that DSp and DMSp, in the absence of SOC channel stimulation, do not have a direct agonist effect on these channels (see Supplementary Fig. 3B–C).

In order to further confirm that the elevated mitochondrial Ca^2+^ observed in deoxysphingoid base-treated MNs is due, at least in part, to dysfunctional SOC entry, FCCP was used to render the mitochondria dysfunctional prior to measuring SOC entry, which prevents mitochondria from contributing to changes in Ca^2+^. FCCP is an uncoupling agent which abolishes the mitochondrial membrane potential by allowing hydrogen ions through the inner mitochondrial space before they can be used for ATP generation by complex V (Benz and McLaughlin, 1983). The results summarised in Fig. 4E, show that when the mitochondrial membrane potential (∆ψ_m_), and therefore active mitochondrial regulation of cytosolic Ca^2+^, is abolished with FCCP, there is an increase in cytosolic Ca^2+^ following opening of SOC channels. This increased cytosolic Ca^2+^ after SOC channel activation was present to similar extents in both vehicle- and DSp- treated MNs, with the fura-2 ratio changing from 0.67 ± 0.05 to 1.11 ± 0.0.13 (*P* < 0.01) in vehicle-treated MNs and from 1.00 ± 0.06 to 1.40 ± 0.11 (*P* < 0.05) in DSp-treated MNs. Application of FCCP led to exacerbated cytosolic Ca^2+^ levels in both vehicle-treated and DSp-treated MNs, providing evidence for an active involvement of mitochondria in Ca^2+^ buffering following SOC channel activation in these cells.

Taken together, these results suggest that a critical effect of deoxysphingoid bases, or their downstream metabolites, may be to alter the cell membrane, which results in dysregulation of SOC channels, which in turn affects mitochondrial Ca^2+^ levels and disturbs ER Ca^2+^.

### 3.10 Deoxysphingoid bases cause mitochondrial dysfunction in motor and sensory cells

**Fig. 5:**
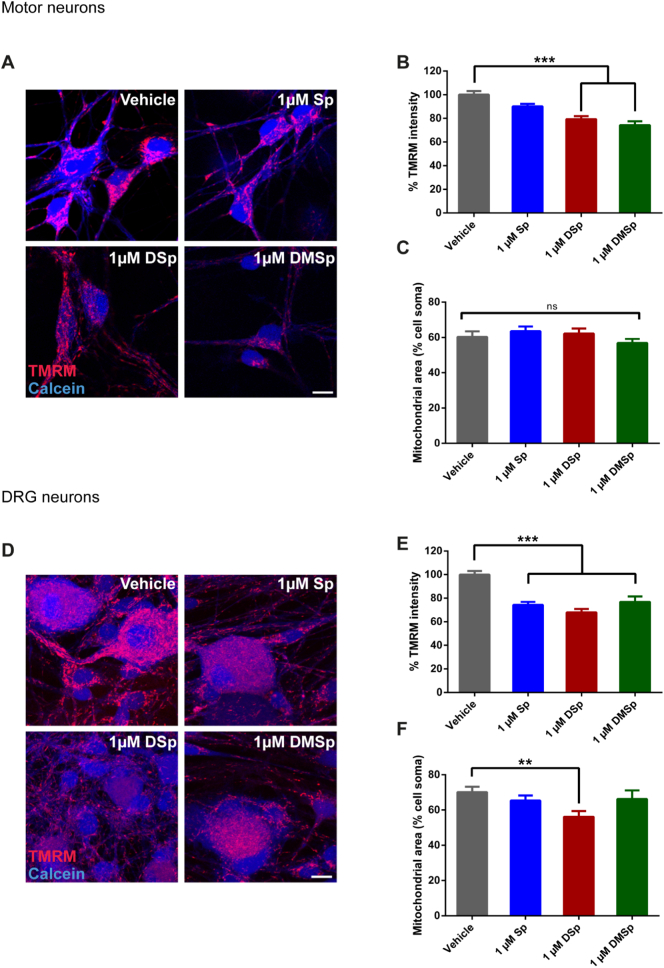
Treatments with deoxysphingoid bases reduce the mitochondrial membrane potential. (A) At 5–8 DIV MNs were treated with sphingoid bases or vehicle control (ethanol) for 2 h prior to live cell imaging. (B) Average TMRM fluorescent intensities per cell were measured from 26 to 48 MNs per condition, from 3 independent experiments. (*P* < 0.001, one-way ANOVA). (C) The total mitochondrial area per MN cell body was also calculated and expressed as a percentage of the cell soma size. (*P* = 0.267, one-way ANOVA). (D) At 3–5 DIV DRG neurons were treated with vehicle control (ethanol) or sphingoid bases 2 h prior to live cell imaging. (E) Average TMRM fluorescent intensities per cell were measured from 29 to 61 cells, from 4 independent experiment. (*P* < 0.001, one-way ANOVA). (F) The total mitochondrial area per DRG cell body was also calculated and expressed as a percentage of the cell soma size. (*P* = 0.017, one-way ANOVA). Error bars represent S.E.M. For statistical comparison, each treatment group was compared to vehicle control. Data was compared to vehicle control using one-way ANOVA and Dunnett's multiple comparisons tests. ns = not significant. *P* values: * < 0.05; ** < 0.01; *** < 0.001. Scale bars = 10 μm.

Having observed mitochondrial Ca^2+^ loading in MNs treated acutely with the deoxysphingoid bases, we next tested whether these treatments also cause a more generalized mitochondrial dysfunction. We investigated changes in mitochondrial membrane potential (∆ψ_m_) following 2 h sphingoid base treatments using TMRM, a cationic dye which accumulates in mitochondria as a function of ∆ψ_m_, in both MN and DRG cultures (Fig. 5A and D, respectively). Following treatment with DSp and DMSp there was a significant decrease in ∆ψ_m_, so that the mean TMRM intensity declined to 79 ± 2% (*P* < 0.001) and 74 ± 3% (*P* < 0.001) of the intensity measured in vehicle treated neurons, respectively (Fig. 5B). In this short 2 h treatment paradigm, the decrease in ∆ψ_m_ was not sufficient to affect the total mitochondrial area, expressed as a percentage of the cell soma size (Fig. 5C).

In parallel, DRG neurons subjected to the same treatment paradigm also displayed a reduced ∆ψ_m_, with DSp-treated DRG neurons having an average TMRM intensity 68 ± 3% of the intensity measured in vehicle-treated DRG neurons (Fig. 5E, *P* < 0.001). DMSp-treated DRG neurons showed a similar decrease in ∆ψ_m_ and intriguingly, even treatment with the typical enzyme product, Sp, caused a depletion in ∆ψ_m_. In these sensory DRG cells, 2 h treatment with DSp was also sufficient to cause a decrease in the mitochondrial area per cell soma, so that DSp-treated DRG neurons showed an average mitochondrial area of 56 ± 3% compared to 70 ± 3% in vehicle-treated DRG neurons (*P* < 0.01, Fig. 5F). The decrease in ∆ψ_m_ observed in these experiments, along with a reduced total mitochondrial area in DRG cells following such short-term treatment with the deoxysphingoid bases, supports the hypothesis that neuronal mitochondria are an early target of deoxysphingoid base-mediated neurotoxicity.

A major pathway for cell death that occurs secondary to mitochondrial dysfunction is the opening of the mitochondrial permeability transition pore (mPTP). The mPTP opens in a bid to release mitochondrial Ca^2+^, which in turn signals cell death *via* the concomitant release of cytochrome C and pro-caspases in order to trigger apoptosis (Susin et al., 1999a; Susin et al., 1999b). To explore whether the opening of the mPTP is responsible for the deoxysphingoid base-mediated neuronal death we observed in this study (Fig. 2), we co-treated MNs with cyclosporine A (CsA), an inhibitor of mPTP opening (Bernardi et al., 1994), alongside the sphingoid bases.

**Fig. 6:**
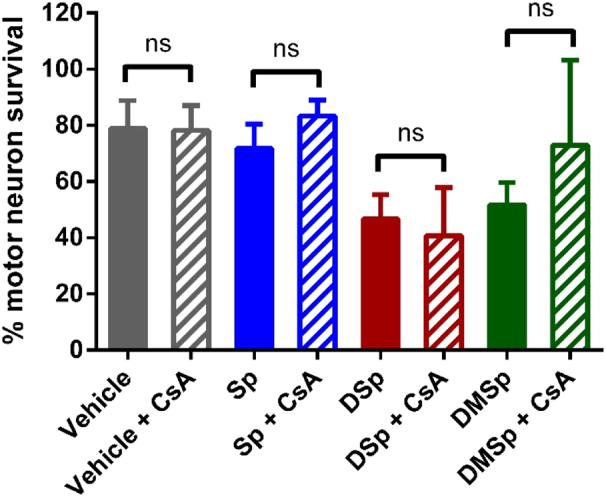
Treatment with cyclosporine A (CsA) is not sufficient to rescue deoxysphingoid base mediated neuronal death. After 24 h *in vitro*, MNs were treated with either Sp, DSp or DMSp alone or in combination with 1 μM CsA. MNs were fixed 24 h following treatment, at 2 DIV, and stained with DAPI and β-III tubulin for analysis. MN survival is expressed as a percentage relative to untreated controls. Statistical analysis was performed using two-way ANOVA: *P* = 0.041, treatment, *P* = 0.491, CsA treatment, *P* = 0.741, interaction. N = 4–10 independent experiments per condition. Error bars represent S.E.M. ns = not significant.

Immature MNs were treated with CsA (1 μM) and the sphingoid bases (1 μM) for 24 h, and cell survival was expressed as a percentage of untreated cultures. There was no significant difference in MN survival in cultures treated with the sphingoid bases alone or with addition of CsA (Fig. 6); 47 ± 9% of MNs survived in 1 μM DSp-treated cultures, compared to 41 ± 17% in DSp + CsA-treated cultures. These results suggest that cell death caused by exogenous application of deoxysphingoid bases is not mediated by mitochondrial permeability transition.

## 4 Discussion

In this study we set out to investigate the intracellular effects of exogenous deoxysphingoid bases, the abnormal enzyme products of mutant SPT in HSN-1, on cultured mammalian neurons. Our results clearly show that deoxysphingoid bases are toxic to primary mammalian neurons. Toxicity by these abnormal sphingoid bases manifests as reduced neurite length and complexity of treated primary MNs (Fig. 1) as well as a dramatic, dose-dependent reduction in cell survival (Fig. 2). We also demonstrated that primary MNs are vulnerable to exogenous application of deoxysphingoid bases both when they are immature and growing, as well as at more mature stages when these cells already have extensive dendritic networks.

The concentration range of the deoxysphingoid bases used in these experiments were conducted within the reported blood plasma levels of deoxysphingoid bases in HSN-1 patients (Auer-Grumbach et al., 2013; Garofalo et al., 2011; Murphy et al., 2013; Penno et al., 2010; Rotthier et al., 2011; Suriyanarayanan et al., 2016). Using these concentrations, the level of toxicity of DSp and DMSp observed in this *in vitro* model of HSN-1 is dramatic, with over 50% of neurons dying after just 1 day of treatment. This is in contrast to the slowly progressing neuropathy observed in HSN-1 patients. However, DSp displayed a more severe pathology than DMSp at the same concentrations tested, as measured by neurite outgrowth assays, which does reflect the higher serum levels of DSp than DMSp measured in patients. Although the primary embryonic MNs used in these experiments may be particularly vulnerable to toxicity, it is possible that *in vivo*, in patients, the blood plasma may act as a “dumping ground” for deoxysphingoid bases and derivatives, whereas levels in the immediate vicinity of neurons might be lower. Moreover, in blood, deoxysphingoid bases are mainly transported on low and very low density lipoproteins and may be complexed with other proteins, which could additionally dampen their toxic effects (Bertea et al., 2010).

In order to elucidate the early, underlying mechanisms involved in the toxicity of deoxysphingoid bases and to identify specific targets, we used short, 2 and 24 h treatment regimens and identified mitochondria and ER as early targets of deoxysphingoid base toxicity.

Although an increase in cytosolic Ca^2+^ is a well-established indicator of cell stress leading to cell death, short-term (2 h) treatment did not result in any change in resting cytosolic Ca^2+^ levels (Supplementary Fig. S4). This suggests that following a 2 h treatment paradigm, MNs were functioning, are able to compensate for any damaging effects of the treatment and are not yet undergoing apoptosis. This experimental model therefore offers an opportunity to probe the underlying mechanisms of deoxysphingoid base toxicity.

The ER is a key organelle for the regulation of cellular Ca^2+^ homeostasis and the SPT enzyme is located in the ER membrane; the ER is thus the site of deoxysphingoid base generation. We observed Ca^2+^ depletion in the ER, indicative of ER stress, as early as 2 h in DRG cells, yet in MNs, which intrinsically appear to store less Ca^2+^ in ER and mitochondria than sensory neurons, there was no measurable depletion of Ca^2+^ in the ER at this time point (Fig. 3C–D). However, ER stress does become apparent at a later stage in MNs, measured in our experiments as a depletion of relative ER Ca^2+^ after 24 h of treatment with the deoxysphingoid bases (Fig. 3B). Our finding that deoxysphingoid bases may affect motor and sensory neurons differentially, or at least the effects may develop on a different time scale, is in line with the clinical manifestation of the disease; typically, HSN-1 patients present with sensory deficits in advance of motor deficits (Reilly, 2007).

Mitochondria also play a major role in buffering cellular Ca^2+^ and mitochondrial abnormalities have been reported in several studies in HSN-1 patient lymphoblasts, as well as in cells treated exogenously with DSp (Alecu et al., 2016b; Myers et al., 2014; Stimpson et al., 2015). Moreover, the mitochondrial outer membrane associates with the ER, where SPT localizes, *via* the mitochondria-associated ER membrane (MAM, Csordas et al., 2006). The MAM is known to play an important role in intermembrane transport of phospholipids as well as a potential role in the transport of ceramide (Hayashi et al., 2009), a downstream component of the *de novo* sphingolipid synthesis pathway, the pathway specifically affected by HSN-1-causing mutations. In this study, 2 h treatment with the sphingoid bases caused a shift towards elevated mitochondrial Ca^2+^ in MNs, but not in sensory neurons (Fig. 3E–F). It could be speculated that elevated mitochondrial Ca^2+^ is a very early event in deoxysphingoid base-mediated toxicity, which precedes depletion of ER Ca^2+^ (seen only after 24 h treatment in MNs), and thus the 2 h treatment model used in these experiments is not sufficiently short to capture changes in mitochondrial Ca^2+^ in DRG cells, in which depletion of ER Ca^2+^ is evident much earlier than in MNs. However, differences in the experimental setup used to measure relative ER Ca^2+^ in DRGs and MNs make it difficult to draw direct comparisons between the two cell types. MNs are developmentally younger when the relative ER Ca^2+^ content is measured as they are derived from embryonic mouse spinal cords, whereas DRG cells are obtained from postnatal mice and this developmental shift might be responsible for the observed differences between relative ER Ca^2+^ measured in motor and sensory neurons. The proposal that mitochondrial Ca^2+^ levels may be affected in advance of ER Ca^2+^ levels mirrors the recent study of Alecu et al. that tracked deoxysphinagnines to the mitochondria within 5 mins of application, and at later time points, also to the ER (Alecu et al., 2016b).

Extracellular treatment of MNs with deoxysphingoid bases leads to a rapid loading of Ca^2+^ into mitochondria, while resting cytosolic Ca^2+^ and relative ER Ca^2+^ are both unaffected following short-term (2 h) treatment. Thus, the most obvious source of Ca^2+^ is the extracellular space through specific cationic channels: voltage-gated channels, second messenger-operated channels, receptor-operated channels, store-operated channels, or under some conditions, the reversal of the sodium-Ca^2+^ exchanger (NCX, Parekh and Putney Jr, 2005). In this study we tested two major groups of cationic channels that could be responsible; activity-dependant voltage-gated ion channels and the store-operated Ca^2+^ (SOC) channels that are regulated by the ER, causing the opening of these channels upon Ca^2+^ store depletion. Membrane depolarization-induced influx of Ca^2+^ was measured after 2 h treatment with sphingoid bases, and no difference was detected between the control and any of the treatment groups, indicating that the deoxysphingoid bases do not directly interfere with this element of normal neuronal activity, at least not at this early phase (Fig. 4A).

Another source of extracellular Ca^2+^ is *via* the SOC channels. The primary role of these channels is to replenish ER Ca^2+^ stocks. A second major destination of Ca^2+^ influx through these channels is mitochondria (Malli et al., 2003) and thus, it is possible that the Ca^2+^ influx induced by opening of SOC channels immediately loads into mitochondria. We observed that a short, 2 h treatment regime with DSp caused a highly significant increase in Ca^2+^ influx through SOC channels (Fig. 4D). Not only did we observe an increase in the amount of Ca^2+^ entry from the extracellular space in DSp-treated cells, but we also frequently observed a fluctuation of Ca^2+^ levels, instead of the relatively smooth decline of Ca^2+^ entry observed in control cells after the initial Ca^2+^ influx (Fig. 4C). Moreover, when mitochondrial Ca^2+^ uptake was abolished with FCCP, we observed an increase in SOC influx into the cytosol in both control and DSp-treated cells (Fig. 4E). Thus, a major destination of Ca^2+^ flowing through the SOC channels in these cells is indeed mitochondria, but in the absence of mitochondrial buffering, Ca^2+^ remains in the cytosol. Taken together, these findings suggest that a source of elevated mitochondrial Ca^2+^ in DSp-treated neurons may be, at least in part, dysfunctional SOC channels. Interestingly, dysregulation of SOC channels has also been implicated in another inherited peripheral neuropathy, Charcot-Marie-Tooth disease, secondary to *GDAP1* mutations (Barneo-Munoz et al., 2015; Gonzalez-Sanchez et al., 2017; Pla-Martin et al., 2013).

Elevated mitochondrial Ca^2+^ levels are damaging to mitochondria and usually result in cell stress and, if the Ca^2+^ loading persists, can lead to apoptosis. In our experiments, elevated mitochondrial Ca^2+^ in MNs was recorded, alongside a reduction in mitochondrial membrane potential, which was displayed in both MNs and DRG neurons, even after only 2 h of treatment with deoxysphingoid bases (Fig. 5B and E). In addition to a depletion of mitochondrial membrane potential, 2 h DSp treatment was also sufficient to cause a decrease in the total mitochondrial area in DRG cells, but not in MNs (Fig. 5C and F). Indeed this provides further evidence that MNs and DRG neurons may respond differently to DSp or DMSp treatments or within different time frames, as is reflected in the clinical progression of HSN-1 in patients where sensory function is affected earlier than motor. Mitochondrial Ca^2+^ loading, as well as loss of mitochondrial membrane potential are known to cause opening of the mitochondrial permeability transition pore (mPTP, Norenberg and Rao, 2007; Szabo and Zoratti, 1992). However, in this study, co-treatment with an mPTP blocker, cyclosporine A (CsA), did not alleviate deoxysphingoid base-mediated neuronal death (Fig. 6). We therefore propose that the elevation in mitochondrial Ca^2+^ observed in this study is a transient change occurring rapidly in response to exogenous deoxysphingoid base application. Thus cell death as a result of deoxysphingoid base application appears here to be independent of mPTP-mediated apoptosis.

DSp, but not DMSp, treatment caused elevated SOC entry in cultured MNs (Fig. 4B) as well as depleted mitochondrial membrane potential and total mitochondrial area in DRG neurons (Fig. 5E–F). This suggests that there may be some functional differences in how DSp and DMSp toxicity manifests, and indeed this may provide reason for the more ready manifestation of DSp toxicity, when compared to DMSp (Fig. 1E–F). There are studies suggesting that neurotoxicity by DSp and DMSp might be mediated by their downstream metabolites (Alecu et al., 2016b; Guntert et al., 2016). In this study we focused exclusively on the physiological effects of extracellular DSp and DMSp application, establishing a link between these abnormal sphingolipids, Ca^2+^ regulation and mitochondrial function.

In conclusion, the results of this study demonstrate that exogenous application of deoxysphingoid bases is toxic to mammalian neurons, manifesting in reduced neurite outgrowth and decreased neuronal survival. Moreover, we also demonstrate that ER and mitochondrial dysfunction may play early roles in deoxysphingoid base-induced neurotoxicity, well before any neuronal death occurs. Depleted ER Ca^2+^ and mitochondrial Ca^2+^ loading, together with a reduction in mitochondrial membrane potential, occur rapidly after treatment with deoxysphingoid bases in cultured neurons. Moreover, the increase in mitochondrial Ca^2+^ loading may be a result of dysregulation of SOC channels located on the cell membrane. As these abnormalities occur at a very early stage, when morphologically these cells appear normal and no cell loss has occurred, it is likely that the observed Ca^2+^ and mitochondrial changes are part of the primary mechanism leading to the eventual neuronal death. Identification of key signalling pathways leading to deoxysphingolipid-mediated neurotoxicity may help reveal targets for therapeutic intervention in HSN-1, in a bid to slow or prevent peripheral nerve damage.

## Acknowledgements & funding

We would like to thank Ione F. G. Meyer, Verna Sarajarvi and James N. Sleigh for their helpful comments during the preparation of this manuscript, James Dick for his excellent technical assistance, and all members of the Linda Greensmith and Giampietro Schiavo laboratories for helpful discussions. We thank Harriet Howard for her help with Western blot procedures. ERW is in receipt of a PhD studentship from the Medical Research Council (MRC) Centre for Neuromuscular Diseases, MRC Centre Grant (G0601943). LG is The Graham Watts Senior Research Fellow supported by The Brain Research Trust. MMR is grateful to the National Institutes of Neurological Diseases and Stroke and Office of Rare Diseases (U54NS065712) for their support. BK was supported by UCLH Charities and the European Community's Seventh Framework Programme (FP7/2007–2013, EUROMOTOR grant No 259867). This work was undertaken at University College London Hospitals/University College London, which received a proportion of funding from the Department of Health's National Institute for Health Research Biomedical Research Centres (BRC51/NS/MR) funding scheme. The content is solely the responsibility of the authors and does not necessarily represent the official views of the National Institutes of Health. Conflicts of interest: none.

## Appendix A Supplementary data

Supplementary material Image 1
