# Supplementary material for: Hereditary sensory neuropathy type 1-associated deoxysphingolipids cause neurotoxicity, acute calcium handling abnormalities and mitochondrial dysfunction in vitro
Source: Neurobiol Dis. 2018 Sep;117:1–14. doi: 10.1016/j.nbd.2018.05.008 (PMC6060082; doi:10.1016/j.nbd.2018.05.008)
Supplement: Supplementary file 1 — Supplementary material [file mmc1.docx]

**Supplementary Figure S1
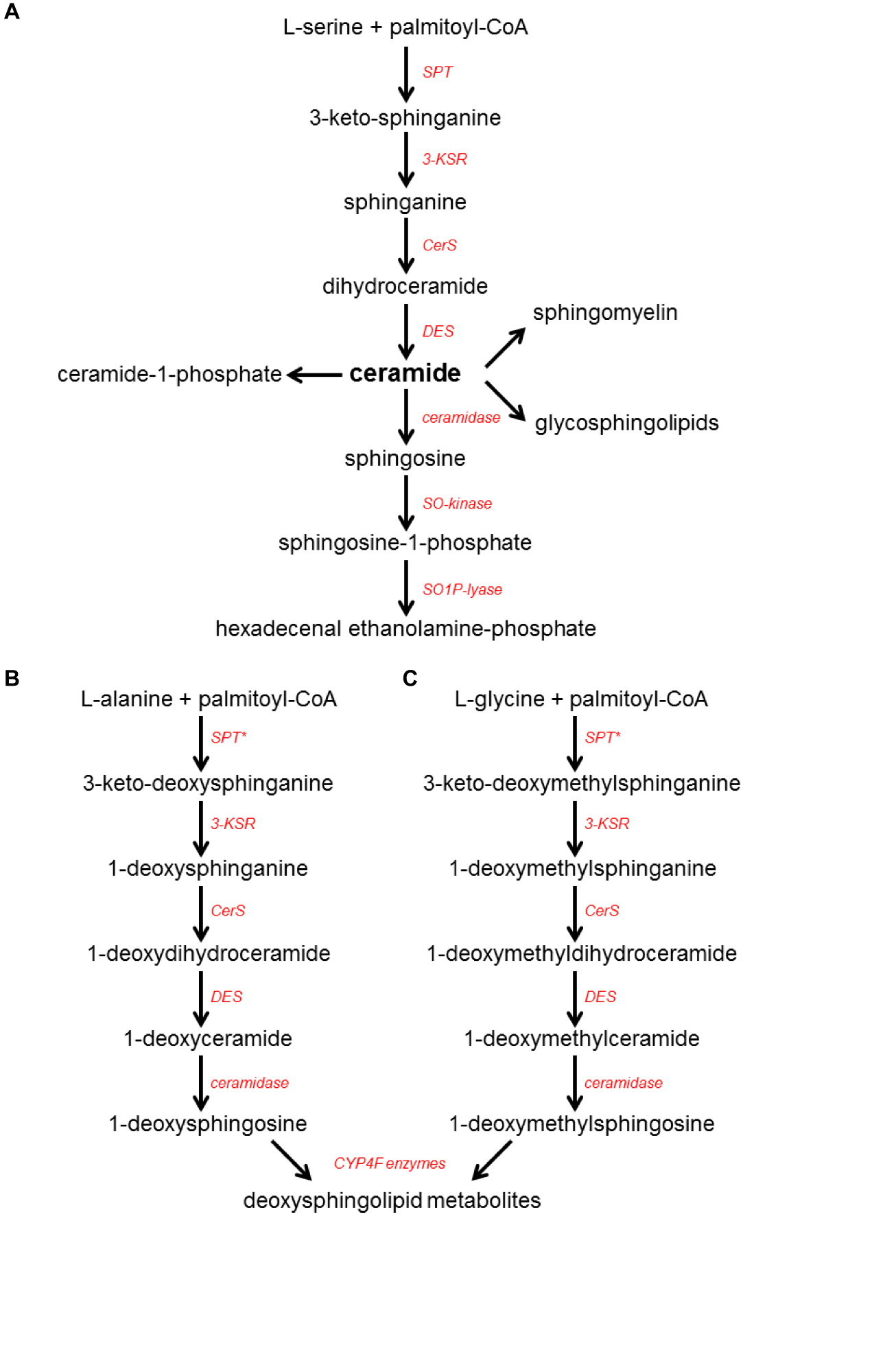
**

# **Supplementary Figure S1: The *de novo* sphingolipid synthesis pathway in mammalian cells**

**(A)** Serine palmitoyltransferase (SPT) catalyzes the initial step of L-serine and palmitoyl coenzyme A condensation to form 3-keto-sphinganine. 3-keto-sphinganine is reduced to sphinganine by 3-keto-sphinganine reductase (3-KSR). Sphinganine is acylated by ceramide synthase (CerS) and subsequently desaturated by ceramide desaturase (DES) to form ceramide. Ceramide is used for the formation of the complex sphingolipids necessary for normal cell function. The degradation of ceramide is by the enzymes ceramidase, sphingosine-kinase (SO-kinase) and sphingosine-1-phosphate lyase (SO1P-lyase), in turn. **(B)** and **(C)** show the intermediate products of the same pathway when mutant SPT (as indicated by an asterix) preferentially binds L-alanine (B) or L-glycine (C), instead of the normal L-serine substrate. Formation of deoxy- species means that normal downstream complex sphingolipids cannot be synthesized. Deoxysphingosines are proposed to be hydroxylated and desaturated into deoxysphingolipid metabolites by the cytochrome P450 4F (CYP4F) subfamily of enzymes (23).

**Supplementary Figure S2**
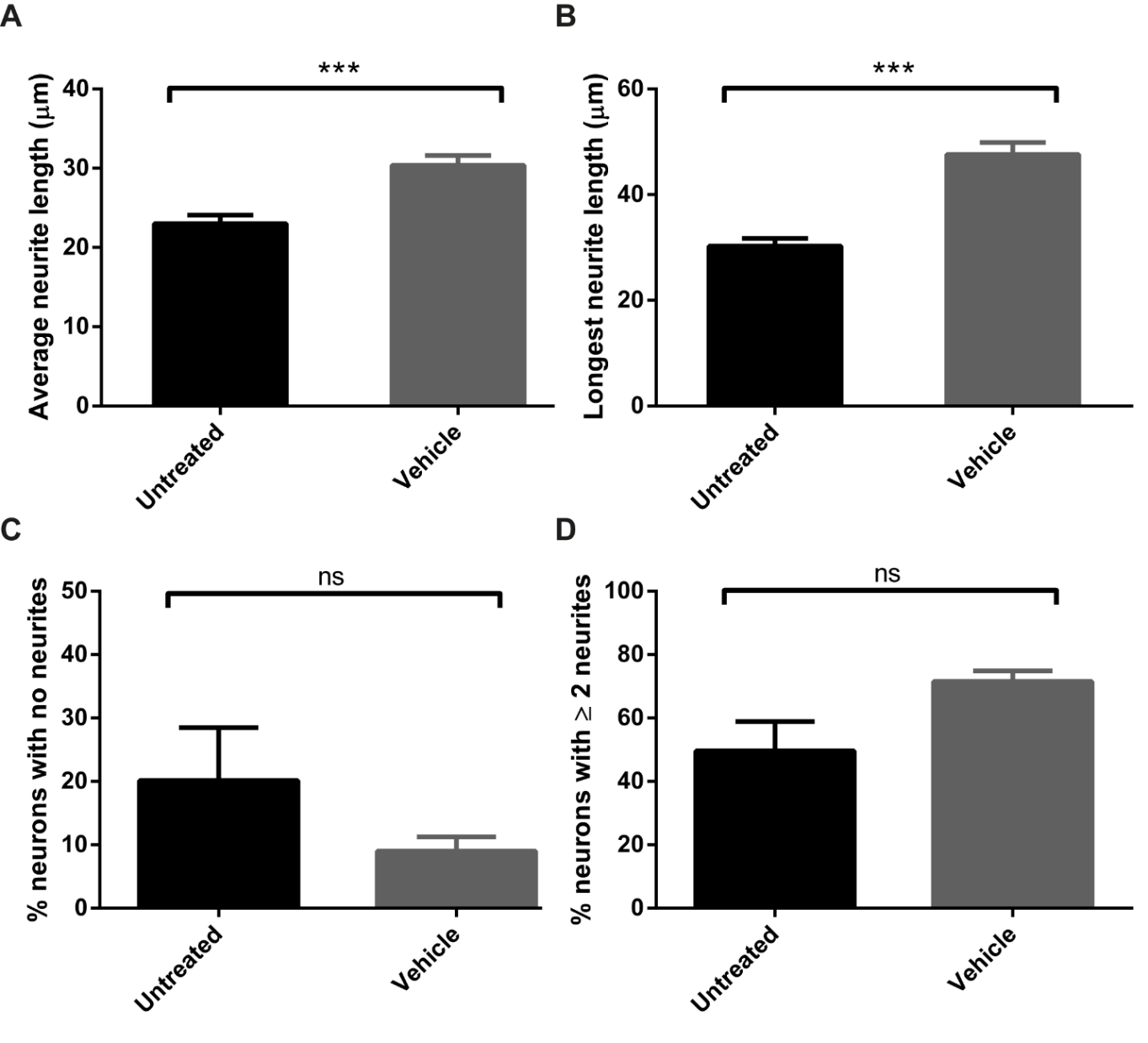


# **Supplementary Figure S2: The effect of an ethanol vehicle control on motor neuron neurite outgrowth**

After 24 h *in vitro*, MNs were treated with ethanol. MNs were fixed and stained for analysis of cell survival, 24 h following treatment, at 2 DIV. Treatment with ethanol caused an increase in **(A)** the average neurite length and **(B)** the length of the longest neurite, per MN, but had no effect on the percentage of MNs with **(C)** no neurites or **(D)** two or more neurites. Error bars represent S.E.M. Pairwise comparisons were made using unpaired t tests or Mann-Whitney tests. *P* values: * < 0.05; ** < 0.01; *** < 0.001. n = a minimum of 383 cells per condition, from 4 independent experiments.

**Supplementary Figure S3**


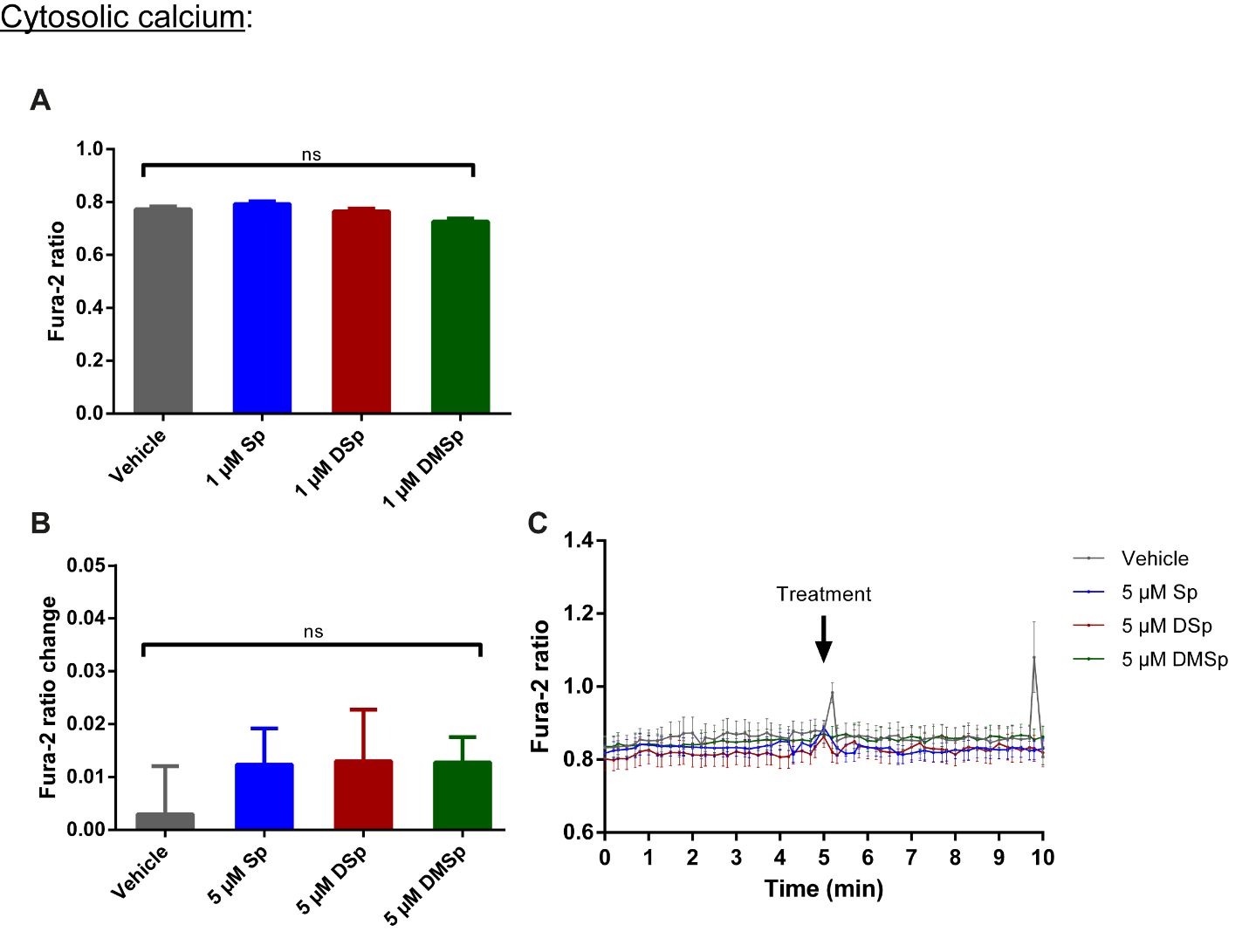


**Supplementary Figure S3: Deoxysphingoid base treatments do not cause a change in cytosolic Ca^2+^**

**(A)** At 5-8 DIV, MNs were treated with either vehicle control (ethanol) or the sphingoid bases, 2 h prior to live cell imaging with fura-2. The average fura-2 ratio, representing cytosolic Ca^2+^ concentration, was established from a minimum of 107 cells per condition, from a minimum of 7 independent experiments. For statistical comparison, each treatment group was compared to vehicle control using Kruskal-Wallis (*P*< 0.001) and Dunn’s multiple comparisons tests. **(B-C)** At 4 DIV, DRGs were treated with either vehicle control (ethanol) or the sphingoid bases during live cell imaging and the fura-2 ratio recorded for 5 min. The average change of fura-2 ratio during the 5 min treatment period was calculated (C), representing cytosolic Ca^2+^ concentration change, from 14-29 cells per condition, from 2 independent experiments. For statistical comparison, each treatment group was compared to vehicle control using Kruskal-Wallis (*P*= 0.9). Error bars represent S.E.M. ns = not significant.

**Supplementary Figure S4**

**
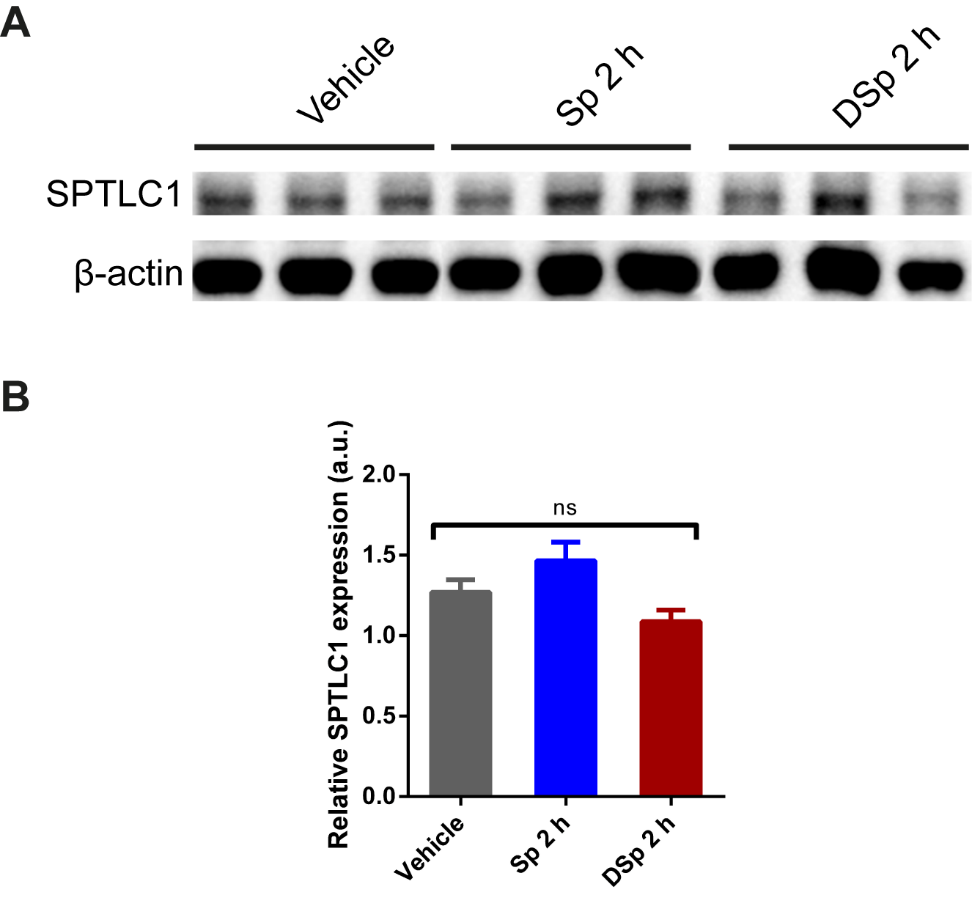
**

# **Supplementary Figure S4: Deoxysphingoid base treatments do not cause a change in SPTLC1 expression**

At 6 DIV, MNs were treated with either vehicle control (ethanol) or the sphingoid bases for 2 h and protein was extracted for analysis. (**A)** Western blot showing the expression of SPTLC1 protein (51 kDa) and the loading control β-actin (42 kDa) from 3 independent experiments. (**B)** The bar chart shows the quantification of SPTLC1 expression normalised to β-actin loading controls, all relative to SPTLC1 expression in the untreated control (not shown). Error bars represent S.E.M. For statistical comparison, each treatment group was compared to vehicle control using one-way ANOVA (*P* < 0.001) and Dunnett’s multiple comparisons tests. ns = not significant.

**Supplementary Materials and Methods**

**Western blot**

For western blot, primary MNs were grown to 6 DIV and treated with sphingoid bases for 2 h before protein extraction. Cells were washed once with ice cold PBS and protein extracted from cells using radioimmunoprecipitation assay (RIPA) buffer containing 50 mM Tris-HCl (pH 7.5), 150 mM NaCl, 1% IGEPAL CA-630, 0.5% sodium deoxycholate, 0.5% sodium dodecyl sulphate, 1 mM EDTA, 1 mM EGTA and Halt protease and phosphatase inhibitor cocktail. Protein concentration per sample was determined using the Bio-Rad *DC* Protein Assay (Bio-Rad Laboratories). Samples were diluted using 4x Laemlli Sample Buffer (Bio-Rad Laboratories) (containing 10% 2-mercaptoethanol) and RIPA buffer, to equal concentrations. Lysates were boiled for 10 min at 95°C in order to denature proteins. Samples were run on a NuPAGE 4-12% Bis-Tris Midi protein gel (1.0 mm, ThermoFisher Scientific) and transferred to a nitrocellulose membrane. The membrane was probed for SPTLC1 (Merck Millipore ABS1642; 1:1000) and β-actin (Abcam ab6276; 1:30000). Protein levels were quantified using ImageLab 5.2.1 software.
